# Dual Biologic Therapy in a Patient With Niemann-Pick Type C and Crohn Disease: A Case Report and Literature Review

**DOI:** 10.1097/PG9.0000000000000225

**Published:** 2022-07-25

**Authors:** Alexandra S. Hudson, Patricia Almeida, Hien Q. Huynh

**Affiliations:** From the Department of Pediatrics, Division of Pediatric Gastroenterology and Nutrition, University of Alberta, Edmonton, Alberta, Canada.

**Keywords:** biologic, case report, dual biologic, genetic disorder, inflammatory bowel disease

## Abstract

Dual biologic therapy has become a new area of interest in inflammatory bowel disease (IBD). Monogenic/polygenic IBD and the role of genetics in IBD is an evolving field, with many of these patients having difficult treatment courses. We present a case of a teenage patient with Niemann-Pick disease type C and Crohn colitis, who sustained clinical remission only after escalating to dual biologic therapy (anti-tumor necrosis factor alpha [infliximab] and anti-interleukin-12/anti-interleukin-23 [ustekinumab]). A literature review of dual biologic therapy in pediatric IBD revealed 8 case series and 1 cohort study. In pediatric patients with genetic disorders and IBD who are not responding adequately to biologic therapy, adding a second biologic medication with a different mechanism of action may be efficacious. Targeting both anti-tumor necrosis factor alpha (which induces pro-inflammatory cytokines) and the pro-inflammatory cytokines themselves (interleukin-12/interleukin-23) may be important in impaired macrophage function and increased cytokine response. Our case adds to the sparse literature on the utility of combining ustekinumab and infliximab in pediatric IBD and is the first to describe its use for treating ongoing active luminal disease.

## INTRODUCTION

The incidence of inflammatory bowel disease (IBD) has been on the rise, currently affecting over 1.5 million individuals in North America and 2 million individuals in Europe (1). As such, research regarding etiology and treatment options has been rapidly expanding, especially in the area of genetic disorders and IBD, given our increasing access to whole genome sequencing. Many monogenetic and polygenetic causes of IBD have been identified (over 150 genetic loci identified to date), but there are many underlying genetic mechanisms that remain elusive (2). Niemann-Pick disease type C (NPC), a neurodegenerative lysosomal storage disorder, is one such genetic disorder, and its’ associated predisposition to IBD is thought to be related to impaired autophagy (3).

Since the availability of biologic and biosimilar medications, IBD therapy has been a rapidly expanding field. Dual biologic therapy has become a new area of interest, particularly in combining biologics with different mechanisms of action, as an attractive option in helping avoid the need for surgery. This is important given that biologic clinical remission rates in Pediatric patients are only approximately 40%–60% at 1 year (4) and that in Canadian children with IBD, 15%–33% will need surgery within 10 years of diagnosis (4–6). It also holds great potential in those who are unlikely to respond to traditional therapy, such as IBD patients with genetic disorders (7). We herein present a case of a teenage patient with NPC and IBD who had sustained clinical remission only after escalating to dual biologic therapy (anti-tumor necrosis factor alpha [anti-TNFα] [infliximab] and anti-interleukin-12/anti-interleukin-23 [ustekinumab]).

## CASE REPORT

A 13-year-old female with a recent diagnosis of NPC presented with a 4-week history of abdominal pain, weight loss, and diarrhea. She had developed bloody and nocturnal stools over the past week. She had no extraintestinal manifestations of IBD. Remote past medical history included a treated stage II Wilms tumor and attention deficit hyperactivity disorder. The family history was negative except for a maternal aunt who was recently diagnosed with ulcerative colitis.

**FIGURE 1.**
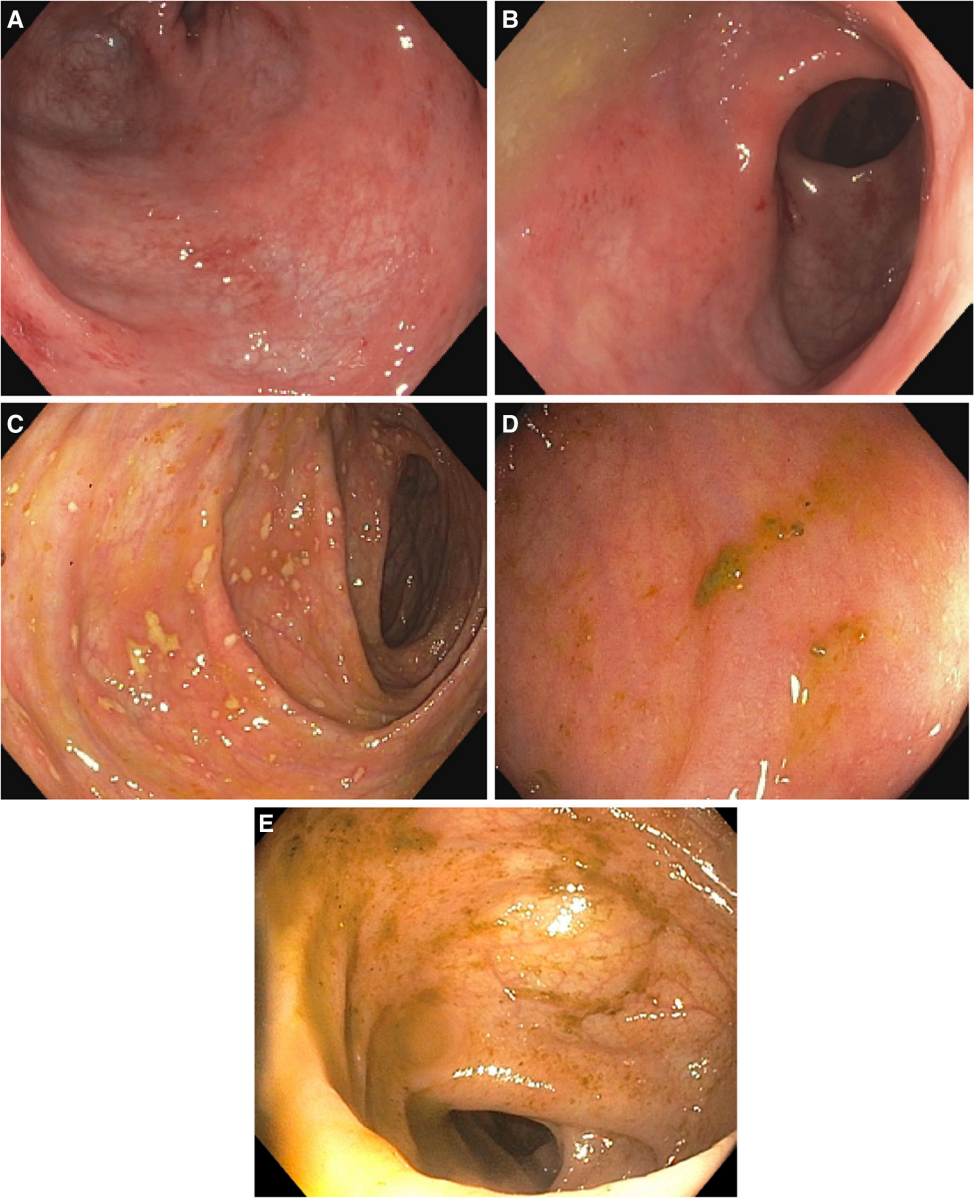
Initial and repeat endoscopic luminal findings. Initial endoscopy showing very mild colitis in the rectosigmoid (A) and rectum (B). C) Repeat endoscopy while on steroids showing new right-sided colitis with aphthous ulcers. D) Repeat endoscopy on infliximab and azathioprine before escalation to ustekinumab showing colitis in the hepatic flexure. E) Repeat endoscopy on dual biologic showing normal rectal mucosa.

Investigations revealed an elevated C-reactive protein (14 mg/L, normal <8.1 mg/L) and erythrocyte sedimentation rate (20 mm/h, normal <10 mm/h) but were otherwise unremarkable, including a negative infectious workup. An esophagogastroduodenoscopy was normal. A colonoscopy revealed very mild inflammation in the rectum and rectosigmoid junction (Fig. 1A, B), with pathology showing mild granulomatous pancolitis (cecum to rectum, with a normal terminal ileum). The Simple Endoscopic Score for Crohn’s Disease total score was 6 (ileum 0, right colon 0, transverse colon 0, left colon 3, rectum 3). Magnetic resonance enterography showed no abnormalities of her small bowel or perianal area.

**FIGURE 2.**
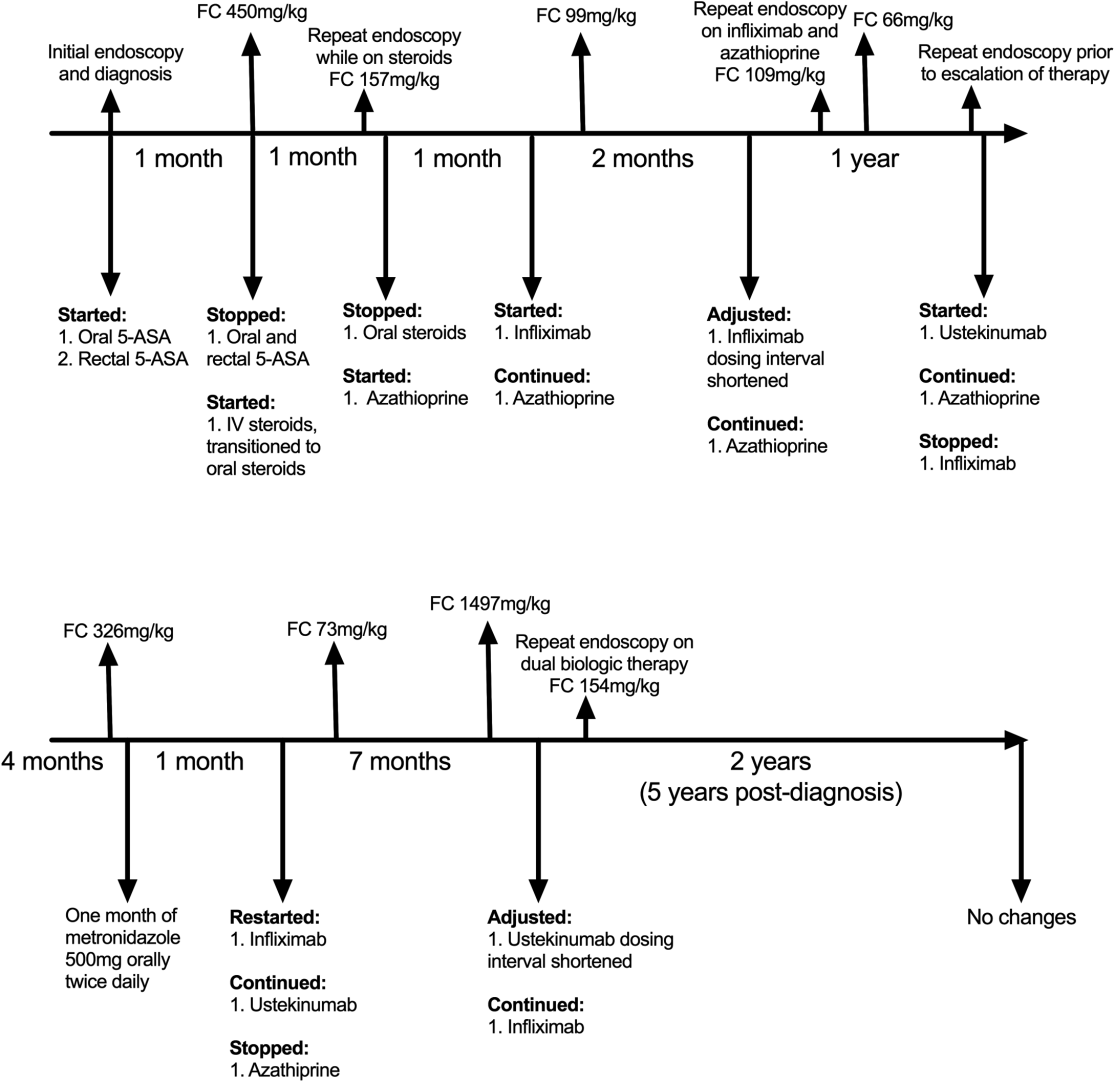
A timeline of medication changes, endoscopy, and fecal calprotectin values. 5-ASA = 5-aminosalicylic acid; FC = fecal calprotectin; IV = intravenous.

A summary of her treatment and endoscopy timeline is displayed in Figure 2. Induction therapy was commenced with rectal (mesalamine 20 mg/kg suppository each evening) and oral 5-aminosalicylic acid (5-ASA) (mesalamine 30 mg/kg BID). Although not typical induction therapy for Crohn’s colitis, given how mild her disease was and after a discussion with the patient and family, 5-ASA was chosen over enteral nutrition, steroids, or an immunomodulator. She had worsening of symptoms and an elevation of fecal calprotectin to 450 mg/kg (normal <50 mg/kg) at 1-month post-diagnosis. Her induction therapy was then changed from 5-ASA to corticosteroids (intravenous [IV] methylprednisolone 0.5 mg/kg q12 h for 3 d, followed by prednisone oral 1 mg/kg daily for 1 mo, then weaning by 5 mg/wk until off).

During her oral steroid wean, her symptoms escalated. Her fecal calprotectin was still elevated at 157 mg/kg. A repeat endoscopy was done, which had worsened, showing mild left-sided patchy colitis and moderate right-sided colitis with aphthous ulcers (Fig. 1C). Pathology corresponded with chronic active colitis in the cecum and right colon and chronic inactive colitis of the left colon and rectum. Given the worsening of her disease, azathioprine (2 mg/kg oral daily) maintenance therapy was added.

Despite the addition of azathioprine, she had little change to her symptoms. The family became more receptive to biologic therapy, so infliximab (10 mg/kg IV at 0, 2, and 6 wk followed by every 6 wk) was added to azathioprine. This demonstrated excellent symptomatic response, with corresponding reduction of her fecal calprotectin to 99 mg/kg. Two months later, she had slightly worsening symptoms and a low infliximab levels (1.7 ug/mL, therapeutic trough level >3 ug/mL), so the dosing interval was shortened (from every 6 wk to every 4 wk). A repeat endoscopy was done 4 months later while on infliximab and azathioprine, showing macroscopically (normal appearance) and microscopic (chronic inactive colitis) remission. The corresponding fecal calprotectin at this time was 104 mg/kg, which decreased even further to 66 mg/kg 3 months later.

One year later, she had a persistent return of symptoms. A repeat endoscopy was done given the likely need to escalate therapy. This showed colitis in the hepatic flexure and the entire left colon to the rectum (Fig. 1D), with pathology demonstrating chronic active colitis with microgranulomas. Infliximab was stopped, and she was started on ustekinumab (2.5 mg/kg IV then 2 mg/kg subcutaneously every 8 wk), with azathioprine ongoing.

After 2 months of ustekinumab with unsatisfactory response, and a rise in fecal calprotectin to 326 mg/kg, a trial of 1 month of metronidazole 500 mg orally twice daily was done, which did not have a symptomatic effect. Therefore, infliximab was readded as a dual biologic (10 mg/kg IV at 0, 2, 6 wk and then every 4 wk). Azathioprine was stopped, and she continued therapy on the 2 biologic medications. This resulted in excellent symptomatic and biochemical response, with near normalization of her fecal calprotectin to 73 mg/kg. She had mild return in her symptoms 7 months into dual biologic therapy, with a rise of her fecal calprotectin to 1497 mg/kg and a lower ustekinumab drug level of 1.161 (therapeutic level 1–10), so the ustekinumab dosing interval was shortened from every 8 weeks to every 4 weeks.

With continuation of this dual biologic therapy with a shortened dosing interval, her symptoms resolved, her fecal calprotectin decreased significantly to 154 mg/kg, and a repeat endoscopy was macroscopically normal (Fig. 1E). The microscopic findings demonstrated a normal terminal ileum and mild active focal colitis in the cecum to rectum. She continued to have ongoing neurological decline, but maintained great symptomatic and biochemical response to the dual biologic therapy 2 years later (5 y post-diagnosis). Fortunately, there were no reported (from patient or parent) adverse effects from any of the medications throughout her treatment course.

## DISCUSSION

Our case adds to the limited available literature on combination biologic therapy in pediatric IBD. Specifically, the majority of cases to date have reported on combining vedolizumab with infliximab or ustekinumab. Therefore, our case adds support to the very limited knowledge around combining ustekinumab with infliximab. Reported pediatric cases to date have also only described the use of ustekinumab with infliximab to treat unwanted side effects of infliximab, not due to active luminal disease. Furthermore, our case provides details of long-term follow-up (2.5 y) after starting dual biologics, of which there is very little known.

The literature on dual biologics in adult IBD is very limited. A 2019 systematic review and pooled analysis of dual biologic therapy in adult patients identified 7 studies (only case reports and case series) using combination therapy with either infliximab and vedolizumab or vedolizumab and ustekinumab in the adult IBD population (8). Focusing on those who received ustekinumab combination biologic therapy (with vedolizumab), there were 3 cases reported (age 22–27 y old), with all patients having significant clinical improvement. Two of the 3 case reports discussed endoscopic findings, which both improved as well. There were no adverse events. The pediatric literature is comparatively even more sparse. The databases PubMed, Scopus, and Google Scholar were searched from inception until November 2021. The following terms (searching all fields) were used to identify articles: “*dual biologic* or *second biologic*” and “*pediatric* or *child*” and “*inflammatory bowel disease* or *IBD* or *crohn* or *ulcerative colitis*.” References of all included articles were also screened for relevant articles. Articles were excluded if they were not written in the English language. Our search identified 2 studies, a 2020 case series (9) and a 2021 cohort study (10). The case series of pediatric IBD patients using combination biological therapy reported 8 patients using infliximab and vedolizumab (50% remission achieved) and 5 patients using infliximab and ustekinumab (100% remission achieved) (9). Of note, these patients combined infliximab and ustekinumab to treat anti-TNFα related psoriasis, not due to their IBD not being in remission. Their literature search also yielded no previous pediatric case reports or case series. They identified an additional 2020 adult patient cases series of ustekinumab combination biologic therapy (with an anti-TNFα in 2 patients); however, they used adalimumab or golimumab as their anti-TNFα (not infliximab), with both patients having a good clinical response (11). The 2021 pediatric cohort study reported 16 children total, with combinations of vedolizumab/tofacitinib/ustekinumab, achieving 75% clinical response (10). There were no combinations of ustekinumab and infliximab.

There is an estimated 7% penetrance of IBD in patients with NPC (3). These patients been shown to have increased susceptibility to fistulizing colitis with granuloma formation, being both early onset and severe, predisposing it to be very difficult to treat (3). This is thought to be due to the impaired destruction of intracellular bacteria within macrophages (3). This persistence of bacteria in the gut wall leads to an increased cytokine response (3). Therefore, any genetic defect in the pathways and signaling cascades of antibacterial autophagy can lead to a Crohn’s disease state. NPC and other genetic disorders with this pathway affected are likely a subset of other monogenic and polygenic causes of IBD. Targeting both anti-TNFα (which induces pro-inflammatory cytokines, among many other roles) and the pro-inflammatory cytokines themselves (interleukin-12/interleukin-23) may be important in genetic disorders with impaired macrophage function and increased cytokine response. Combining medications that target gut wall cytokines in multiple mechanisms in these difficult-to-treat patients, can lead to synergistic effects, improving the efficacy of treatment.

Dual biologic therapy for medically refractory IBD is an important evolving field in IBD, with especially limited knowledge thus far for the pediatric IBD population. Pediatric patients with concurrent genetic disorders and IBD may be particularly at risk of developing difficult-to-treat IBD and may benefit from the addition of a second biologic medication with a different mechanism of action. This is a potential option even several years into treatment. To date, only 1 pediatric case study and 1 pediatric cohort study are available, with the majority combining vedolizumab with infliximab. Our case adds to the sparse literature on the utility of combining ustekinumab and infliximab, and to our knowledge is the first case to describe the use of this combination in controlling active luminal IBD. This is an area that warrants further research. Limitations of our study include the nature of it being a case report with experience of only 1 patient and its retrospective nature, including missing data.

## ACKNOWLEDGMENTS

A.S.H. involved in substantial contributions to the design of the work; acquired, analyzed, and interpreted data for the work; drafted the work; revised it critically for important intellectual content; gave final approval of the version to be published; and agrees to be accountable for all aspects of the work in ensuring that questions related to the accuracy or integrity of any part of the work are appropriately investigated and resolved. P.A. involved in substantial contributions to the conception and design of the work; acquired data for the work; drafted the work; gave final approval of the version to be published; and agrees to be accountable for all aspects of the work in ensuring that questions related to the accuracy or integrity of any part of the work are appropriately investigated and resolved. H.Q.H. involved in substantial contributions to the conception and design of the work; interpreted data for the work; revised it critically for important intellectual content; gave final approval of the version to be published; and agrees to be accountable for all aspects of the work in ensuring that questions related to the accuracy or integrity of any part of the work are appropriately investigated and resolved.

## References

[1] Ng SC Shi HY Hamidi N. Worldwide incidence and prevalence of inflammatory bowel disease in the 21st century: a systematic review of population-based studies. Lancet. 2018;390:2769–2778. 10.1016/S0140-6736(17)32448-0 29050646

[2] Uhlig HH. Monogenic diseases associated with intestinal inflammation: implications for the understanding of inflammatory bowel disease. Gut. 2013;62:1795–1805. 24203055 10.1136/gutjnl-2012-303956

[3] Schwerd T Pandey S Yang HT. Impaired antibacterial autophagy links granulomatous intestinal inflammation in Niemann-Pick disease type C1 and XIAP deficiency with NOD2 variants in Crohn’s disease. Gut. 2017;66:1060–1073. 26953272 10.1136/gutjnl-2015-310382 PMC5532464

[4] Nasiri S Kuenzig ME Benchimol EI. Long-term outcomes of pediatric inflammatory bowel disease. Semin Pediatr Surg. 2017;26:398–404. 29126510 10.1053/j.sempedsurg.2017.10.010

[5] Kuenzig ME Benchimol EI Lee L. The impact of inflammatory bowel disease in Canada 2018: direct costs and health services utilization. J Can Assoc Gastroenterol. 2019;2(suppl 1):S17–S33. 31294382 10.1093/jcag/gwy055 PMC6512251

[6] Carroll MW Kuenzig ME Mack DR. The impact of inflammatory bowel disease in Canada 2018: children and adolescents with IBD. J Can Assoc Gastroenterol. 2019;2(suppl 1):S49–S67. 31294385 10.1093/jcag/gwy056 PMC6512244

[7] Kanegane H. Inflammatory bowel diseases and primary immunodeficiency diseases. Immunol Med. 2018;41:154–161. 30632919 10.1080/25785826.2018.1556025

[8] Ribaldone DG Pellicano R Vernero M. Dual biological therapy with anti-TNF, vedolizumab or ustekinumab in inflammatory bowel disease: a systematic review with pool analysis. Scand J Gastroenterol. 2019;54:407–413. 30945576 10.1080/00365521.2019.1597159

[9] Olbjørn C Rove JB Jahnsen J. Combination of biological agents in moderate to severe pediatric inflammatory bowel disease: a case series and review of the literature. Paediatr Drugs. 2020;22:409–416. 32378002 10.1007/s40272-020-00396-1 PMC7383034

[10] Dolinger MT Spencer EA Lai J. Dual biologic and small molecule therapy for the treatment of refractory pediatric inflammatory bowel disease. Inflamm Bowel Dis. 2021;27:1210–1214. 33125058 10.1093/ibd/izaa277

[11] Kwapisz L Raffals LE Bruining DH. Combination biologic therapy in inflammatory bowel disease: experience from a tertiary care center. Clin Gastroenterol Hepatol. 2021;19:616–617. 32068149 10.1016/j.cgh.2020.02.017

